# Preventing (impulsive) errors: Electrophysiological evidence for online inhibitory control over incorrect responses

**DOI:** 10.1111/psyp.12647

**Published:** 2016-03-23

**Authors:** Borís Burle, Wery P. M. van den Wildenberg, Laure Spieser, K. Richard Ridderinkhof

**Affiliations:** ^1^ Aix‐Marseille Université, CNRSLNC UMR 7291MarseilleFrance; ^2^ Department of PsychologyUniversity of AmsterdamAmsterdamThe Netherlands; ^3^ Amsterdam Brain & Cognition (ABC)University of AmsterdamAmsterdamThe Netherlands

**Keywords:** Response inhibition, Simon task, EEG, Current source density

## Abstract

In a rich environment, with multiple action affordances, selective action inhibition is critical in preventing the execution of inappropriate responses. Here, we studied the origin and the dynamics of incorrect response inhibition and how it can be modulated by task demands. We used EEG in a conflict task where the probability of compatible and incompatible trials was varied. This allowed us to modulate the strength of the prepotent response, and hence to increase the risk of errors, while keeping the probability of the two responses equal. The correct response activation and execution was not affected by compatibility or by probability. In contrast, incorrect response inhibition in the primary motor cortex ipsilateral to the correct response was more pronounced on incompatible trials, especially in the condition where most of the trials were compatible, indicating a modulation of inhibitory strength within the course of the action. Two prefrontal activities, one medial and one lateral, were also observed before the response, and their potential links with the observed inhibitory pattern observed are discussed.

In a rich environment, with multiple action affordances, it is not only essential to select and execute the most appropriate action, but preventing the execution of inappropriate actions is also critical. In this respect, inhibitory control plays a key role in adaptive action selection. Understanding the dynamics and the origin of incorrect response inhibition, and how it can be modulated by task demands, is the primary goal of this study. Neurophysiological measures have implicated both medial (e.g., Duque, Olivier, & Rushworth, 2013) and lateral (e.g., Aron, Robbins, & Poldrack, 2004, 2014) prefrontal areas in inhibitory control (see also Filevich, Kühn, & Haggard, 2012; Ridderinkhof, Forstmann, Wylie, Burle, & van den Wildenberg, 2011, for reviews), and showed that such inhibitory control acts at the level of the primary motor cortices (Burle, Bonnet, Vidal, Possamaï, & Hasbroucq, 2002; Duque et al., 2013; Leocani, Cohen, Wasserman, Ikoma, & Hallett, 2000; Thura & Cisek, 2014). These regions will be the main focus of the present report, and their activity will be tracked with EEG to reach an adequate temporal resolution.

The dynamics and the role of response inhibition in response selection is at the core of formal decision‐making models that aim at describing how we decide between and select appropriate actions—one of the key questions in experimental psychology and cognitive neurosciences. A general framework that explains such action decisions is provided by sequential sampling model of decision making (see Bogacz, Brown, Moehlis, Homes, & Cohen, 2006; Ratcliff & McKoon, 2008, for overviews). In this general framework, it is assumed that one accumulates evidence in favor of each response alternative over time, and that as soon as one has received enough evidence, the corresponding action is issued. This type of model was originally designed to account for choice behavior, and has been extremely successful in accounting for performance (including accuracy and response time) in a large number of tasks in humans (Ratcliff & McKoon, 2008). Although extremely powerful, such a behavioral fitting approach in humans does not allow for access of the latent assumptions of the models or of their internal dynamics (Servant, White, Montagnini, & Burle, 2015). In the last few years, single‐cell recordings in monkeys have provided detailed description of the underlying decision dynamics: when monkeys have to decide the global motion direction of a noisy cloud of points, movement neurons in the lateral intraparietal cortex (LIP) show a pattern of activity that is highly consistent with the core assumptions of sequential sampling models (see Gold & Shadlen, 2007, for an overview). Neurons whose receptive field corresponds to the end point of the saccade corresponding to the dominant direction show a progressive increase in firing rate until a given value is reached, and the saccade executed. More importantly for the current goal, the firing rate of neurons whose receptive field corresponds to the incorrect saccade decreases as time passes, indicating that deciding indeed entails an inhibitory component.

While these models are widely used to fit behavioral performance, neurophysiological investigations in humans are much more scarce. One powerful approach, though, has been to combine fMRI data with the best‐fitting model parameters to get insight into which brain areas BOLD signals covary with the estimated parameters (Forstmann, Dutilh et al., 2008; van Maanen et al., 2011; see Mulder, van Maanen, & Forstmann, 2014, for an overview). High temporal resolution techniques, such as EEG or magnetoencephalography (MEG) allow us to more directly study the development of evidence accumulation. Only few studies have used these measures to probe decision‐making models, which mainly focused on occipital and parietal activity that seem to correspond to information accumulation (O'Connell, Dockree, & Kelly, 2012; Philiastides, Ratcliff, & Sajda, 2006; Ratcliff, Philiastides, & Sajda, 2009; see Kelly & O'Connell, 2015, for a recent overview). Interestingly, a MEG study (Donner, Siegel, Fries, & Engel, 2009) has reported that such perceptual evidence accumulation appears to flow down to the primary motor cortices (M1s), where activities in the beta and gamma range covary with evidence accumulation.

However, the use of conventional EEG (and, to a much lower extent, of MEG) has a main limitation. Because of volume conduction (Nunez & Westdorp, 1994; Tenke & Kayser, 2012), the scalp‐recorded potentials are a mixture of the underlying cortical activities (Makeig, Bell, Jung, & Sejnowski, 1996). This mixture distorts the recovered scalp signals both in space and time (Burle et al., 2015), making it difficult to establish “linking propositions” (Teller, 1984) between observed potentials and decision‐making processes (Vidal et al., 2015). The use of current source density (through surface Laplacian [SL] estimation) has proved to be very efficient at removing the noncortical‐induced volume conduction. Indeed, being proportional to the radial component of the cortical current density, SL removes the noncerebral volume conduction effect, and hence provides a fair approximation of the corticogram, that is, the activity one would record if electrodes were positioned on the surface of the cortex. As a consequence, it significantly improves the spatial resolution of EEG from 9–10 cm to 2–3 cm (Gevins, 1989; Nunez & Srinivasan, 2006; see Kayser & Tenke, 2015, for a recent overview). This spatial resolution increase is exemplified in Figure 1. This figure shows the same data (grand average of all conditions mixed of the data reported below) at response time before (panels A and C) and after (panels B and D) SL computation. Thanks to this spatial improvement, it is possible to dissociate the activity of the left and right primary motor cortices (M1) when tasks require subjects to select between a right‐ and a left‐hand response (Praamstra & Seiss, 2005; Tandonnet, Burle, Vidal, & Hasbroucq, 2003; Vidal, Grapperon, Bonnet, & Hasbroucq, 2003; van de Laar, van den Wildenberg, van Boxtel, & van der Molen, 2011). SL reveals a negative wave over the M1 contralateral to the response (i.e., involved in the execution of the correct response) that starts about 70 ms before electromyogram (EMG) onset and reaches its maximum around EMG onset. Symmetrically, a positive wave develops simultaneously over the M1 ipsilateral to the response (i.e., involved in the not‐to‐be issued, or incorrect, response). Note that one can generally not map negativity and positivity to either activation or inhibition, since the scalp polarity depends not only on the nature (excitatory or inhibitory) of the underlying synaptic activity, but also on the cortical layer in which this synaptic activity is generated (see, e.g., Westbrook, 2000). However, in the present case, the negativity occurring over the contralateral M1 very likely reflects the activation of this cortex. As a matter of fact, M1 is an agranular cortex, without layer IV (see, e.g., Amunts, Schleicher, & Zilles, 2007). As a consequence, there are no deep afferences, and the synaptic activity one records with EEG mainly arises from superficial layers. The contralateral negativity thus reflects superficial excitatory postsynaptic potentials (EPSP). Symmetrically, the positivity over the M1 contralateral to the incorrect response cannot be a deep EPSP; hence, it reflects a superficial inhibitory postsynaptic potential. Furthermore, in the same time windows (a few tens of milliseconds before EMG onset), direct measures of cortical (Burle, Bonnet et al., 2002; Duque et al., 2013; Tandonnet, Garry, & Summers, 2011; Verleger, Kuniecki, Möller, Fritzmannova, & Siebner, 2009) and corticospinal (Hasbroucq, Akamatsu, Burle, Bonnet, & Possamaï, 2000) excitability have shown that, while the motor structures involved in the correct response get more and more excitable toward response execution, the excitability of the structures involved in the incorrect response decreases, indexing without ambiguity an inhibition of the incorrect response. For these reasons, the ipsilateral positivity can be safely interpreted as the electrophysiogical correlate of the incorrect response inhibition observed with stimulation techniques (see Burle, Vidal, Tandonnet, & Hasbroucq, 2004, for a review and argumentation). The temporal dynamics of this pattern nicely resemble the firing rate pattern of movement neurons in LIP for saccadic movements (see, e.g., Roitman & Shadlen, 2002), making it a potentially powerful marker of response evidence accumulation in bimanual choice reaction time (RT) tasks.

**Figure 1:**
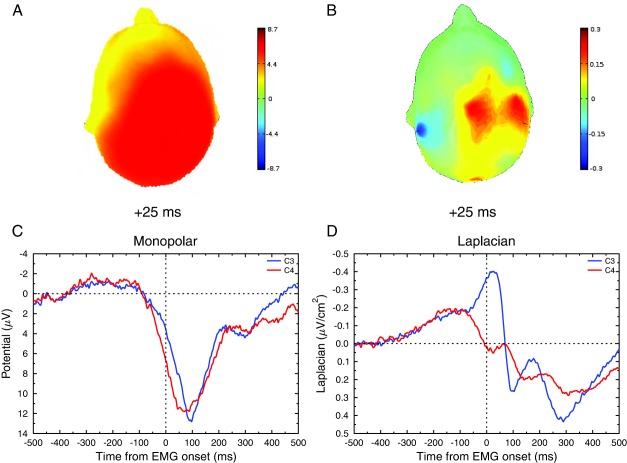
Example of the effect of SL transform on EEG spatial resolution. (A) and (C) show the topography (25 ms after EMG onset) and the time course (C3 and C4) of the monopolar data, respectively. Topography indicates a widespread positivity, whose time course is very similar across electrodes. (B) and (D) show the same data after SL transformation. The topography (25 ms after EMG onset) now clearly shows a negativity over C3 and a positivity over C4. The time courses confirm a very different evolution of C3 and C4 activities around EMG onset (D).

This activation/inhibition pattern is of particular interest since it provides a unique window on cortical control over response selection and execution. We took advantage of this activation/inhibition pattern to gain knowledge on the psychological and brain processes underlying simple decision making. Of particular interest are the origin and the modulation of the inhibition of incorrect response.

In formal models of decision making, response inhibition can take two main forms (Bogacz et al., 2006): it can be mutual or feedforward. Mutual inhibition refers to the notion that each response inhibits its competing response(s) proportionally to its own activation (Usher & McClelland, 2001), whereas feedforward (or proactive) models state that activation and inhibition of response come from upstream evidence (Heuer, 1987). At an abstract level of description, in both types of model, one can write that activation *A* of response *i*( $A_{i}$) at time *t*+1 is equal to

(1) $$A_{i}(t+1)=A_{i}(t)+I_{i}+W_{i}‐\alpha_{i}Inhibition(t)$$

where $\alpha_{i}$ corresponds to a parameter modulating the inhibition received by response *i*, $I_{i}$ is the amount of evidence supporting response *i*, and $W_{i\;}$is random white noise. When inhibition is equal to activation, both models reduce to a particular model—the drift diffusion model (Bogacz et al., 2006; Ratcliff & Smith, 2004). In this case, activation and inhibition are perfectly anticorrelated.

Empirically, however, neuron firing rates for correct and incorrect responses are not perfectly anticorrelated (see, e.g., Roitman & Shadlen, 2002). In humans, the contralateral negativity and ipsilateral positivity are only partially related, and it has been proposed that the incorrect response inhibition also entails an active error‐prevention process (Burle et al., 2004). In agreement with this view, inhibition was absent, but activation was preserved when the to‐be‐given response was known in advance, such that participants only had to decide to respond or not (go/no‐go task; Vidal, Burle, Grapperon, & Hasbroucq, 2011). Biasing the probability of responses also dissociated activation and inhibition: when a highly probable response turned out to be the incorrect response alternative, the associated M1 was very strongly inhibited while the correct response activation was not affected (Meckler et al., 2010). Although these two datasets are in agreement with an error‐prevention mechanism, the way inhibitory control is modulated remains open. Indeed, in both cases, the critical factor was manipulated blockwise; it could be that the strength of inhibitory control was set a priori. For example, in the go/no‐go task, the to‐be‐given response being known in advance, one can imagine that α is simply a priori set to zero. In the response probability bias situations, the most likely response also being known, it could be that α value is set higher for the likely response, so that when the low probability response is activated, the amount of inhibition of the most likely response is very high to compensate the probability bias. Alternatively, the α values could be modulated dynamically, within the course of the action, to adapt to the context, indicating a highly flexible act of control. Evaluating whether incorrect response inhibition can also be modulated online within the course of the action according to the context was the first goal of this study.

Besides revealing this activation/inhibition pattern over the M1s, SL also evidenced medial and lateral prefrontal activities occurring before or around M1 activations (Roger, Bénar, Vidal, Hasbroucq, & Burle, 2010; Vidal et al., 2003). Since both medial (e.g., Duque et al., 2013) and lateral (e.g., Aron et al., 2004, 2014) prefrontal areas have been proposed to have a critical role in inhibitory control (see also Filevich et al., 2012; Ridderinkhof et al., 2011, for reviews), we investigated the link between those areas and the incorrect response inhibition.

To evaluate whether the strength of the incorrect response inhibition can be modulated online, within the course of a trial, we recorded EEG activity in a Simon task, in which the proportion of incompatible trials was biased. In some blocks, most of the trials were incompatible and relatively few were compatible (INC/com context), whereas in other blocks, relatively few trials were incompatible and most trials were compatible (COM/inc context) (Logan & Zbrodoff, 1979; see also Jáskowski, Skalska, & Verleger, 2003, for an EEG investigation with subliminal cues). In this last condition, response activation triggered by stimulus position is assumed to be stronger (Ridderinkhof, 2002), increasing the necessity of inhibiting the ispilateral response to prevent incorrect responses. Note that manipulating the probability of compatible and incompatible trials allows us to keep equal the probability of the two motor responses. As a consequence, participants cannot anticipate which response hand should be inhibited and hence cannot a priori bias their inhibitory control. For this reason, any observed modulation of the inhibition will necessarily reflect online adaptation (see below).

## Method

### Participants

Twelve young adults (age range 18–37, 6 females) participated in the experiment. Some of them were first year psychology students at the University of Amsterdam and received course credit for their participation. The others were junior researchers at the Psychology Department of the University of Amsterdam. All were naive regarding the goals of the experiment, and all gave written informed consent. All procedures were performed in accordance with relevant laws and institutional guidelines.

### Apparatus and Stimuli

Subjects were seated in a dimly lit, sound‐attenuated experimental room, facing a computer screen (refresh rate 100 Hz). A small white square (1.6 × 1.6 mm) presented at the center of the screen served as fixation. The stimuli were colored circles (8 mm in diameter) presented either above or below fixation at a distance of 6.6 mm from fixation. The circles could be filled in red (RGB code: 255,0,0), in green (0,255,0) or in blue (0,0,255).

Two plastic cylinders (3 cm in diameter, 7.5 cm height) were fixed centrally in front of the subject, so that their arms were forming an angle of 90° when holding them. One was close to the subject (the down button) and the other was more distant, fixed 20 cm in front of the first one (the up button). On the top of these cylinders, force sensors (Interlink Electronics force sensitive resistors, 24 mm in diameter) were glued and served as response devices. Responses were issued by the thumb of the right and left hand. For a response to be recorded, the force had to exceed 20 N (Newtons), which corresponds to 2 kilograms‐force, that is, a force corresponding to a weight of 2 Kg.

### Task and Procedure

Subjects were instructed to respond as fast and accurately as possible according to the color of the stimulus. One color was associated with the up response (e.g., red), one color with the down response (e.g., green), and the remaining one (e.g., blue) served as no‐go signal. The color‐to‐response mapping was counterbalanced across subjects. The position of the stimulus was entirely irrelevant for the task at hand. However, the signal could appear in the same vertical hemifield as the response (e.g., the red stimulus, calling for an up response, was presented above fixation—called the *compatible* trial), or in the opposite vertical hemifield (e.g., the red stimulus presented below fixation—called the *incompatible* trial). Obviously, no‐go trials could not be classified as compatible or incompatible.

Half of the subjects responded with the left hand using the up button (and the right hand using the down button), whereas the reverse hand position was used for the other half. This counterbalancing was mixed with the color‐to‐response mappings. There were thus 12 combinations, and each subject was assigned to one of these combinations.

Each trial started with the onset of the fixation point in the center of the screen. After 500 ms, the stimulus was presented and remained on the screen for 800 ms, irrespective of the participant's RT, which was also the time allowed for the response to be given. After this interval, the screen went blank for 800 ms and a new trial was started. The intertrial interval was thus 2.1 s. Subjects first performed a training session without EEG recordings. During this training session, subjects first performed a block of 40 trials, with go compatible trials only, to practice the color‐to‐response mapping and to get familiar with the amount of force to produce for the response to be recorded. Thereafter, they continued the practice session by running four blocks of 42 trials containing one third of compatible trials, one third of incompatible trials, and the last third of no‐go trials.

The experimental session was scheduled on a different day and consisted of 24 blocks of 60 trials. Two types of blocks were defined. In one type of block, the proportion of trials was as follows: 70% compatible, 20% incompatible, and 10% no‐go (COM/inc context). In the other type of block, the proportion of compatible and incompatible was reversed: 20% compatible, 70% incompatible, 10% no‐go (INC/com context). These two probability conditions were administered in four series of six blocks. Half the subjects started with six COM/inc blocks, followed by six INC/com ones, which were followed again by six COM/inc and six INC/com blocks. The other half of the subjects performed the blocks in the opposite order.

### Electrophysiological Recordings

The EEG was recorded with QuickCap (NeuroScan) containing 34 Ag/AgCl electrodes positioned at Fp1, Fp2, AFz, Fz, F1, F2, F3, F4, FCz, FC1, FC2, FC3, FC4, FT7, FT8, Cz, C1, C2, C3, C4, C5, C6, CPz, CP1, CP2, CP3, CP4, Pz, P1, P2, P7, P8, POz, Oz. The electrode configuration corresponds to a density of 64 electrodes over the regions of primary interest (central and frontocentral) and a lower density over temporal and posterior regions to better constrain the interpolation. The reference and the ground were located on the left and right mastoids, respectively. Electrooculogram (EOG) was recorded bipolarly between an electrode above the left eye and an electrode located on the left canthus. The EEG and EOG signals were filtered between 0.1 and 100 Hz. To better synchronize EEG activity with the motor response, EMG activity was recorded by means of Ag/AgCl electrodes attached about 2 cm apart on the thenar eminence above the muscles primarily involved in thumb flexion (flexor pollicis brevis). The EMG signals were filtered between 10 Hz and 500 Hz. EEG electrode impedance was kept below 5 kΩ. All the signals were recorded by SynAmps amplifiers (NeuroScan). The sampling frequency of both EEG and EMG was set at 1000 Hz. Both the EMG and EEG were carefully monitored online, and, as soon as the EMG became noisy, the experimenter instructed the subject to relax his/her hand muscles, to facilitate EMG onset detection. The force signals were recorded by an independent acquisition line with a sampling frequency also set at 1000 Hz.

### Data Processing

All chronometric data were submitted to Student *t* tests when implying only two conditions, and to analysis of variance (ANOVA) otherwise. In such cases, the error term was always the interaction between participants and the factor under analysis. When appropriate, Greenhouse‐Geisser correction for sphericity is reported. Proportions cannot be submitted to ANOVAs as their mean and variance are closely related (Winer, 1971). The arcsine transform has proved to be efficient in stabilizing the variance. All the proportions were therefore corrected before being analyzed.

### Signal Processing

#### EMG

Although automated algorithms speed up the detection of EMG onset, visual inspection is more accurate (Staude, Flachenecker, Daumer, & Wolf, 2001; van Boxtel, van der Molen, Jennings, & Brunia, 2001). Therefore, all individual trials were visualized, and the onset of the EMG activities was marked on a trial‐by‐trial basis. Note that the experimenter was not aware of the nature (compatible vs. incompatible) of the trials during the marking procedure. Go trials were labeled as correct or error depending on whether the correct or the incorrect hand first crossed the force threshold, respectively. In the present report, we focused on correct trials in which only one EMG burst was present on the correct muscles (*pure‐correct* trials). All trials not corresponding to this pattern were discarded, including when a small incorrect EMG burst was observed before the correct one (termed *partial‐error* trials, see Burle, Possamaï, Vidal, Bonnet, & Hasbroucq, 2002; Burle, Spieser, Servant, & Hasbroucq, 2014, for examples). This resulted in about 72% of the data kept for analysis. Likewise, no‐go trials were labeled as *false alarm* if one of the two response buttons reached the force threshold, and *correct omission* if not. Note that, by definition, all responses are erroneous as no‐go stimuli require no overt response. Furthermore, there are no compatible or incompatible no‐go trials. In order to evaluate the stimulus position effect, we classified no‐go trials depending on whether the false alarm was issued with the hand corresponding or not corresponding to stimulus position.

#### EEG

Ocular artifacts were subtracted by a statistical method (Gratton, Coles, & Donchin, 1983). If the subtraction was not judged satisfactory, the artifact was rejected by visual inspection of the monopolar recordings. All the traces were visually inspected, and artifacts were manually rejected. Considerable care was taken to reject local, even small, artifacts, as the SL computation is very sensitive to such local artifacts. The signals were segmented offline and averaged time‐locked to the correct EMG onset. SL computation was performed using Brain Analyzer software. First, the signal was interpolated with the spherical spline interpolation procedure of Perrin, Pernier, Bertrand, and Echallier (1989), and subsequently the second derivatives in the two dimensions of space were computed. We chose three as the degree of spline since this value minimizes errors (Perrin, Bertrand, & Pernier, 1987), and the interpolation was computed with a maximum of 15 degrees for the Legendre polynomial. We assumed a radius of 10 cm for the sphere rather than the implicit unrealistic default radius of 1 m implemented in Brain Analyzer. With such a radius, the most suitable unit is μV/cm2. Figure 1 shows EMG‐locked activities, before and after SL computation. For additional analyses, the data were imported into EEGLAB (Delorme & Makeig, 2004), and custom processing was performed using Python (www.python.org).

We measured the area under the signal in predefined time windows and the slope of the component of interest, which is independent of baseline. The slopes were computed by fitting a linear regression to the signal in the window of interest. To improve the reliability of measures, the data were collapsed across response sides: the left electrode activities for right‐hand responses were averaged with the right electrode activities for left‐hand responses, and symmetrically, left electrodes for left‐hand responses were averaged with right electrodes for right‐hand responses. Therefore, when referring to odd electrodes, we in fact refer to the activity of the hemispheres contralateral to the correct response (i.e., involved in the correct response), and when referring to even electrodes, we refer to the electrodes above the hemisphere contralateral to the incorrect responses (i.e., involved in the incorrect response).^1^ We used the SL activity obtained over C3 and C4 for assessing correct response activation and incorrect response inhibition.

## Results

We will first present an overview of the behavioral findings. Next, we will analyze the activation and inhibition of responses recorded above the M1s. Finally, we will analyze activities recorded above medial and dorsolateral prefrontal cortex and occurring before response.

### Behavioral Measures

The behavioral data are presented in Table 1. The analysis of choice error rates revealed that subjects made more errors in the COM/inc blocks than in the INC/com ones, *F*(1,11) = 13.61; *p* < .004. There were also more errors on incompatible trials than on compatible ones, *F*(1,11) = 36; *p* < .001. The factors compatibility and context interacted significantly, *F*(1,11) = 19.94; *p* < .001, indicating that the difference in error rates between compatible and incompatible trials was more pronounced in the COM/inc context. As a matter of fact, there was no compatibility effect in the INC/com condition, *F*(1,11) = 1.25; *p* = .29, while it was clearly significant in the COM/inc condition, *F*(1,11) = 69.93; *p* < .001.

**Table 1:** Behavioral Results

	Context			
	INC/com		COM/inc	
Go trials	Compatible	Incompatible	Compatible	Incompatible
Overall RT	419	426	393	454
Pure‐correct RT	407	411	385	437
% choice errors	3.36	3.72	1.36	10.07
No‐go trials	Contralateral	Ipsilateral	Contralateral	Ipsilateral
% Commission error	4.28	2.89	2.31	7.52

On no‐go trials, the percentage of false alarms (i.e., responses to no‐go stimuli) followed a pattern similar to choice errors (see Table 1, bottom rows). However, due to the low number of no‐go trials, the overall number of commission errors was too low for a reliable analysis (too many cells were equal to zero).

Overall analysis of response speed revealed no main effect of context on RT, *F*(1,11) < 1, but yielded a clear effect of compatibility, *F*(1,11) = 179; *p* < .001. RT was globally longer on incompatible trials than on compatible trials. Context and compatibility interacted significantly, *F*(1,11) = 31.93; *p* < .001. Planned comparisons revealed no significant compatibility effect in the INC/com context, *F*(1,11) = 1.88; *p* = .19. Conversely, in the COM/inc context, incompatible responses were markedly slower than compatible responses, *F*(1,11) = 115; *p* <.001.

### Correct Response Activation

**Figure 2:**
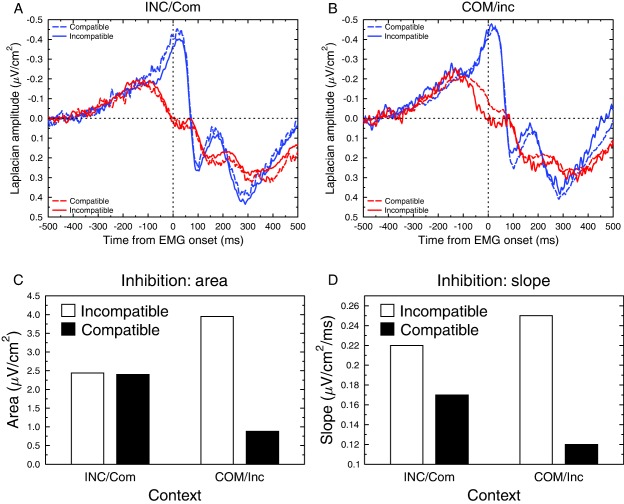
Activity recorded over the M1s, time‐locked to EMG onset. A: Time course of response activation (blue lines) and inhibition (red lines) for compatible (dotted lines) and incompatible (solid lines) trials for the INC/com situation. B: Time course of response activation (blue lines) and inhibition (red lines) for compatible (dotted lines) and incompatible (solid lines) trials for the COM/inc situation. C: Mean area under the ipsilateral positivity for compatible (black) and incompatible (white) as a function of the context. D: Mean slope of the ipsilateral positivity for compatible (black) and incompatible (white) as a function of the context.

We analyzed the negativity obtained contralateral to the correct response (blue lines in Figure 2A,B). We first performed an area analysis (from −75 to 25 ms, baseline from −150 to −100, just before the onset of the phasic negative component). This analysis revealed no main effect of context, *F* < 1, a trend toward a compatibility effect, *F*(1,11) = 3.59; *p* = .085, and no interaction between these two factors, *F* < 1. To clarify the marginal effect of compatibility, and the potentially distorting impact of baseline on such an effect, we also performed a slope analysis (from −100 to 0). The two factors (context and compatibility) were far from significant (both *F*s < 1), and so was the interaction, *F*(1,11) = 1.65; *p* = .23. Overall, these analyses suggest no effect of context or compatibility on the negativity recorded contralateral to the correct response. Since this negativity likely represents the building up of the central motor command, this indicates that, at the cortical level, response execution processes are not affected by compatibility, paralleling the absence of compatibility effects on the motor time (separating EMG onset from mechanical response; Burle, Possamaï et al., 2002; Hasbroucq, Possamaï, Bonnet, & Vidal, 1999; Rösler & Finger, 1993).

### Incorrect Response Inhibition

We shall now focus on the inhibition part, that is, the positive‐going wave recorded over the hemisphere involved in the not‐to‐be‐executed (incorrect) response (red lines in Figure 2A,B). We performed an area analysis in a window from −100 ms before EMG onset to EMG onset (time 0, baseline −150 to −100, Figure 2C). This analysis revealed no main effect of context, *F* < 1, but a main effect of compatibility showed up, with a more pronounced positivity on incompatible trials, *F*(1,11) = 5.9; *p* < .05. The interaction Compatibility × Context was significant, *F*(1,11) = 5.30; *p* < .05, indicating that there was no effect of compatibility on inhibition in the INC/com context, *F* < 1 (Figure 2A,C), but a significant one in the COM/inc context, *F*(1,11) = 12.25; *p* < .005 (Figure 2B,C). This compatibility effect in the COM/inc context shows an increased positivity recorded over M1 involved in the incorrect response for incompatible compared to compatible trials when incompatible trials were rare. Although according to standard statistical theory an interaction should be qualified with only one set of contrasts to avoid reuse of variance, the second contrast was requested during the review process and is thus provided here. It revealed that context effect was significant on incompatible trials, *F*(1,11) = 5.11; *p* < .05, and marginally significant for compatible trials, *F*(1,11) = 3.26; *p* = .10.

In order to avoid any spurious baseline effect, this area analysis was complemented by slope analyses between −100 and 0 ms (see Figure 2D): The slope of this positive wave was steeper overall for the incompatible than for the compatible trials, *F*(1,11) = 4.88; *p* < .05, and there was no difference between INC/com and COM/inc contexts, *F* < 1. Although the interaction was not significant, *F*(1,11) = 2.40; *p* = .15, given the results obtained in the area analyses, we performed the same planned comparisons as for the area analysis reported above. This analysis revealed that the compatibility effect was present in the COM/inc context, *F*(1,11) = 6.78; *p* < .03 (Figure 2B,D), but absent in the INC/com context, *F*(1,11) = 1.58; *p* = .23 (Figure 2A,D), thus confirming the effects obtained with area analysis, even if this latter slope analysis should be taken with caution, given the absence of interaction.

Data obtained over M1s revealed that the incorrect prepotent responses are more inhibited. Since the prefrontal cortices (medial and lateral) have been proposed to play a critical role in inhibitory processes, we search for potential markers of the agent of this inhibition.

### Medial Prefrontal Activity

**Figure 3:**
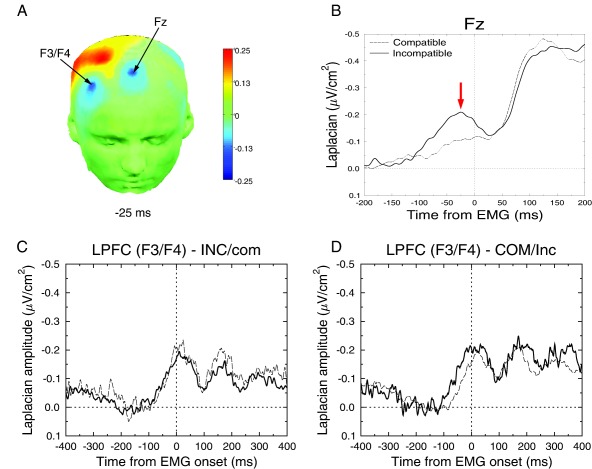
Activities obtained over prefrontal activities, time‐locked to EMG onset. A: Topographies (Laplacian) of the medial and lateral activities. The color bar is in $\mu V/{cm}^{2}$. B: Time course of the medial activity for incompatible (thick line) and compatible (thin line) trials. C: Time course of lateral prefrontal activity in the INC/com situation for the incompatible and compatible trials (same code as in B). D: Time course of lateral prefrontal activity in the COM/inc situation for the incompatible and compatible trials (same code as in B).

Preceding the primary motor cortices activity, a frontocentral activity (over FCz) has been reported in choice RT tasks (Carbonnell et al., 2013; Vidal et al., 2011, 2003). This activity, termed *N*−40, has been proposed to be involved in response selection (Vidal et al., 2003) and has been implicated in the inhibition of the incorrect response (Burle et al., 2004). Figure 3 presents a similar activity, with a maximum of activity on Fz (panel A), and a time course showing a peak just before EMG onset (panel B). As Vidal et al. (2003) reported that this activity preceded the activity of the M1s, we tested whether this was also the case in the present data. To do so, we computed the slopes of the activity over Fz in several time windows, from −200 ms to 0 with a 50‐ms step (see Table 2) to measure when this activity starts. We then estimated when these slopes deviate significantly from zero (one‐tailed *t* test, correction for multiple comparisons assessed by Holm's test; Holm, 1979). As Table 2 indicates, at least 150 ms before EMG onset, the slope significantly differs from zero for incompatible trials, but it never does so on compatible trials (see Figure 3B). Since, as shown in Figure 2, the differential activation of the two M1s (i.e., when contra‐ and ipsilateral M1s start to dissociate) does not occur before −100 ms, the Fz activity starts before response activation and inhibition. We further estimated whether it also peaks earlier. We thus estimated the peak latency of the Fz and C3 activity around EMG onset. This analysis revealed that Fz peaks earlier (−23 ms) than C3 (+17 ms), *F*(1,11) = 46.93; *p* < .0001. No effect of context was obtained, *F*(1,11) = 1.78; *p* = .21, nor any interaction between electrode (C3 vs. Fz) and context, *F* < 1. Since no reliable activity was present on compatible trials, it was not possible to assess the compatibility effects on the latency of this component. In contrast, the compatibility effect could be studied using the area under the curve. Statistical analyses were performed in a window from −100 to 0 ms (baseline from −300 to −100 ms). The analysis revealed an effect of compatibility, *F*(1,11) = 6.47; *p* < .03, but no effect of context, *F*(1,11) = 1.77; *p* = .21. The interaction between these two factors was not significant, *F*(1,11) = 1.75; *p* = .21.

**Table 2:** Slopes and Associated T Values of Activity Above Fz for Compatible and Incompatible Trials

	Compatible				Incompatible			
	Slope	*t* value	*p* value	Holm sign	Slope	*t* value	*p* value	Holm sign
COM/inc								
−200/−150	−0.022	−0.49	.32	*n.s*.	0.081	1.13	.14	*n.s*.
−150/−100	−0.031	−0.94	.18	*n.s*.	−0.118	−2.36	.019	*
−100/−50	−0.123	−1.22	.12	*n.s*.	−0.244	−2.54	.014	*
−50/0	−0.025	−0.60	.28	*n.s*.	−0.003	−0.03	.49	*n.s*.
INC/com								
−200/−150	−0.086	−2.13	.028	*n.s*.	−0.061	1.43	.09	*n.s*.
−150/−100	−0.058	−1.18	.13	*n.s*.	−0.093	−1.80	.049	.
−100/−50	−0.128	−1.49	.08	*n.s*.	−0.183	−5.03	<.001	*
−50/0	−0.068	−0.62	.27	*n.s*.	−0.001	−0.02	.49	*n.s*.

*Note*. Slopes are in $\mu V/{cm}^{2}/ms$. The slope values are given, along with their associated *t* value and their corresponding significance. Since negative slopes are expected, one‐tailed *t* tests were used. To take into account multiple comparisons, the Holm correction was applied (Holm sign). *n.s*. = not significant; . = marginally significant; * = significant at .05.

### Lateral Prefrontal Activities

The SL analyses further revealed a lateral activity, maximal over F3/F4, that can be clearly dissociated from the Fz activity (see Figure 3A).^2^ This activity appears ipsilateral to the inhibited (incorrect) response (although it appears on F4 on the mirrored data, we verified that this activity was present on F4 for a right‐hand response and on F3 for a left‐hand response). We measured the area of this activity in the same time window as for Fz activity, that is, from −100 to 0 ms (baseline from −500 to −350). As lateral EEG signals are often affected by muscular activity on frontal electrodes, such EMG activity was removed by a canonical correlation analysis (De Clercq, Vergult, Vanrumste, Van Paesschen, & Van Huffel, 2006; De Vos et al., 2010). The analysis revealed a main effect of compatibility, *F*(1,11) = 6.26; *p* < .03, and no main effect of context, *F* < 1. The interaction Compatibility × Context was marginally significant, *F*(1,11) = 3.97; *p* = .07. Planned comparisons revealed that the effect of compatibility was absent in the **INC**/**com** context, *F* < 1, but clearly significant in the **COM**/**inc** context, *F*(1,11) = 14.61; *p* < .003. To be sure, the same analysis over the contralateral electrodes revealed no significant effect (all *F*s < 1).

As for Fz, we estimated when slope of the ipsilateral activity significantly differs from zero, for each condition. The results are given in Table 3. Globally, this activity starts to differ from −100 ms to 0 before EMG onset.

**Table 3:** Slopes and Associated T Values of Activity Above F3/F4 for All Experimental Conditions

	Compatible				Incompatible			
	Slope	*t* value	*p* value	Holm sign	Slope	*t* value	*p* value	Holm sign
COM/inc								
−200/−150	0.04	1.26	.23	*n.s*.	0.011	0.22	.82	*n.s*.
−150/−100	−0.032	−1.01	.16	*n.s*.	−0.008	−0.15	.44	*n.s*.
−100/−50	−0.123	−2.83	.008	*	−0.192	−2.93	.006	*
−50/0	−0.216	−4.87	<.001	*	−0.179	−2.19	.025	*
INC/com								
−200/−150	0.073	1.10	.29	*n.s*.	−0.015	−0.38	.35	*n.s*.
−150/−100	0.038	0.88	.40	*n.s*.	−0.097	−3.00	.006	*
−100/−50	−0.164	−2.56	.013	*	−0.157	−3.77	.001	*
−50/0		−0.329	−6.71	< .001	*	−0.147	−1.91	.04

*Note*. Slopes are in $\mu V/{cm}^{2}/ms$. The slope values are given, along with their associated *t* value and their corresponding significance. Since negative slopes are expected, one‐tailed *t* tests were used. To take into account multiple comparisons, the Holm correction was applied (Holm sign). *n.s*. = not significant; . = marginally significant; * = significant at .05.

## Discussion

Behaving adaptively in an environment full of tempting but inappropriate action opportunities is often considered to require active inhibitory mechanisms to avoid incorrect actions to be committed. Although such inhibition of incorrect responses is now well established (Burle, Bonnet et al., 2002; Duque et al., 2013; Tandonnet et al., 2011; Vidal et al., 2003), how this inhibition is implemented remains controversial (Aron et al., 2014). One remaining question is whether this inhibition can be transiently strengthened online to meet changes in inhibitory demands. Here, we addressed this critical question by manipulating the strength of the prepotent response, without a priori biasing the left or right response hand. This was done through a visual Simon task in which the probability of compatible and incompatible trials was manipulated between blocks of trials.

### Incorrect Response Inhibition

Separating the activity of the two M1s allowed quantifying incorrect response inhibition and its potential modulation by the context. While the amount of inhibition was very similar for compatible and incompatible trials in the INC/com condition, it dramatically differed in the COM/inc one: inhibition was enhanced on incompatible trials and possibly reduced on compatible ones. It is interesting to note that the EEG traces are highly comparable across experimental conditions, and differ only during a very limited time window that corresponds to the inhibition period (see Figure 2B). As context effect depends on trial compatibility, it is hence not due to a global change in inhibitory control, but rather reflects stronger specific inhibition. As confirmed by a larger behavioral compatibility effect in the COM/inc context, stimulus position more strongly activates the ipsilateral response, increasing the likelihood of triggering an error on incompatible trials. To override this risk of error, the ipsilateral response appears more strongly inhibited. Symmetrically, the incorrect response on compatible trials, much less likely to trigger an error, appears less inhibited. This modulation of inhibition cannot be set a priori as participants could not anticipate which response would have to be inhibited, hence this modulation necessarily occurred after stimulus presentation. This indicates that inhibitory control can be adjusted online, reactively, within the course of the action, to meet the current demands. Additionally, while inhibition was modulated by context, activation was not, showing a dissociation between these two phenomena. Previous studies also reported a dissociation between response activation and inhibition (Meckler et al., 2010; Tandonnet, Burle, Vidal, & Hasbroucq, 2006; Vidal et al., 2011). However, in these previous reports, the nature of the manipulation allowed the inhibitory parameters to be strategically set a priori (it could have been asymmetrical in the probability bias condition, and set to zero in the go/no‐go). Such an a priori setting cannot account for the dissociation between response activation and inhibition shown in the present study (Figure 2), and the incorrect response inhibition cannot be a mere consequence of correct response activation. Altogether, to the best of our knowledge, these results are the first demonstration of such a fast, highly specific modulation of inhibitory control to prevent impulsive errors.

One may argue that an alternative account of the results might be that, early during processing, both responses are aspecifically activated. Once the correct response has been selected, this nonspecific activation turns specific, and the incorrect response activation simply starts receding. However, the direct prediction of such passive interruption of activation is that the corticospinal excitability of the incorrect motor structures simply goes back to a baseline level. Although this is difficult to assess with EEG, stimulation studies have shown that such excitability does not simply return to baseline, but actually goes below baseline (Burle, Bonnet et al., 2002; Duque et al., 2013; Hasbroucq et al., 2000). Furthermore, it is difficult to see why such a passive interruption would be faster and steeper on some conditions. It seems here necessary to call for an active inhibitory mechanism, and not simply a passive “return to baseline.”

### Upstream Areas Controlling Response Activation and Inhibition?

Modulations of inhibition strength occurred independently of activation modulations, arguing against a mutual inhibition hypothesis, hence favoring the idea of feedforward inhibition. In these lines, online inhibitory modulations could be controlled by upstream structures involved in response control (Burle et al., 2004; Duque, Labruna, Verset, Olivier, & Ivry, 2012; Duque et al., 2013). Both the lateral (Aron et al., 2004; Duque et al., 2012; Neubert, Mars, Buch, Olivier, & Rushworth, 2010) and the medial (Burle et al., 2004; Duque et al., 2013; Mars et al., 2009) parts of prefrontal cortex (LPFC and MPFC, respectively) have been shown to be implicated in inhibitory control (for an overview, see Ridderinkhof et al., 2011).^3^ A recent meta‐analysis (Filevich et al., 2012) of so‐called negative motor areas (NMAs) provides evidence that NMAs in humans largely cluster in the medial (supplementary motor area [SMA] and/or pre‐SMA), and in the lateral parts of the prefrontal cortex (including the inferior frontal gyrus). Their respective roles, however, remain to be specified. Some authors have proposed that these two regions, in combination with basal ganglia nuclei (such as the subthalamic nucleus, STN), form an interacting inhibitory network (see, e.g., Aron et al., 2004; Forstmann, van den Wildenberg, & Ridderinkhof, 2008; Swann et al., 2012), with MPFC mediating the connection between LPFC and STN (Duann, Ide, Luo, & Li, 2009). Recent data, in contrast, suggest that the MPFC and LPFC act independently on STN (Herz et al., 2014). It has also been suggested that these two prefrontal regions of the prefrontal cortex may be involved in different types of inhibition, namely, internally versus externally triggered inhibition, respectively (Duque et al., 2012; Filevich et al., 2012), although Schel et al. (2014) recently showed the LPFC to be activated in both types of inhibition. This network has so far been mainly investigated with transcranial magnetic stimulation (TMS) or fMRI, and hence mainly with limited temporal resolution.^4^ Although TMS studies have provided hints concerning the relative timing of those areas (Neubert et al., 2010), the time course of their activity still remains poorly understood in humans (see Swann et al., 2012, for some chronometry). By improving the spatial resolution of EEG, we could recover both medial (at electrode Fz) and lateral (at electrodes F3/F4, ipsilateral to the incorrect, inhibited M1) prefrontal activities prior or during response execution and inhibition (Figure 3). Interestingly, the pattern of activity on these two sets of electrodes differs and may provide useful clues on their respective roles.

First, the activities recorded over MPFC and LPFC differed in timing with respect to M1 activities. Whereas activity over LPFC was concomitant with M1 activity, activity over MPFC started before the ones over LPFC and M1, as already reported (Vidal et al., 2003). Such a precedence of the MPFC seems in disagreement with the notion that LPFC has its inhibitory influence via connections involving MPFC (Duann et al., 2009), since a reversed timing would be expected. It is, however, compatible with a hierarchical organization between MPFC and LPFC (Neubert et al., 2010; Swann et al., 2012) or with parallel processing routes (Herz et al., 2014).

Besides timing, comparing modulation of response inhibition and of prefrontal activities also provides interesting cues. Activity over MPFC was overall larger for incompatible trials, as was the incorrect response inhibition. However, while the incorrect response inhibition was qualified by an interaction between compatibility and context (incorrect response inhibition was larger for incompatible trials only in the COM/inc context), the interaction was not significant for Fz activity, suggesting that the increase of incorrect response inhibition was not accompanied by an increased activity in the mediofrontal cortex. This partial covariance, while not excluding the role of MPFC in the direct control of response inhibition, suggests that MPFC might not be solely responsible for M1 inhibition. In contrast, the modulation of activity over LPFC nicely parallels the context dependency of incorrect response inhibition, suggesting that it might be directly involved in controlling this process. However, the timing of activity over LPFC coincided with the emergence of the ipsilateral positivity recorded over M1s, whereas one would expect that an area at the origin of the inhibition should be activated earlier to ensure timely inhibition.

One way to reconcile these data patterns is to assume that the two inhibitory sources combine. MPFC, while engaging in the response selection process (Carbonnell et al., 2013; Vidal et al., 2011), would start to override the incorrect response as soon as the correct response has been selected, especially when such selection is difficult. Slightly later, the LPFC might come into play, possibly recruited by MPFC as suggested by EEG functional connectivity analysis (Cohen & Ridderinkhof, 2013), and further strengthen inhibition when needed. The net inhibition would thus be the sum of the two parallel inhibitory routes, stemming from MPFC and LPFC. This proposition is in line with recent data (Herz et al., 2014), suggesting that MPFC and LPFC may have independent influences on M1s, potentially through the basal ganglia.

In conclusion, we demonstrated for the first time a specific, within‐trial and transient increase in the strength of response inhibition. This transient increase reflects an active control mechanism to suppress an incorrect prepotent response, and would be under control of the prefrontal cortex, both its medial and lateral parts, which work in concert to finely regulate inhibitory control.

## Footnotes

1 For all the analyses reported, we also checked that the pattern of results was the same for the two response hands.

2 Inspection of Figure 3A may suggest that this activity is due to a dipolar activity originating from more posterior regions. This is, however, not the case, since the time course of the negative and positive activities are completely different.

3 Both in MPFC and LPFC prefrontal cortices, different subregions have been described, and the respective roles of these subregions is also a matter of debate. However, even after SL transform, the spatial resolution of EEG does not allow us to dissociate these subregions; hence, we will simply refer to them as MPFC versus LPFC.

4 By itself, TMS has an excellent temporal resolution. However, since one probes the excitability at discrete points in time, the actual temporal resolution of the reconstructed activation curve is limited by the temporal sampling of TMS pulses.
